# HIV Risk-Reduction Counseling and Testing on Behavior Change of MSM

**DOI:** 10.1371/journal.pone.0069740

**Published:** 2013-07-29

**Authors:** Xiping Huan, Weiming Tang, Giridhara R. Babu, Jianjun Li, Min Zhang, Xiaoyan Liu, Hongjing Yan, Gengfeng Fu, Jinkou Zhao, Haitao Yang, Roger Detels

**Affiliations:** 1 Jiangsu Provincial Central for Disease Prevention and Control, Nanjing, Jiangsu, China; 2 Department of Epidemiology, School of Public Health, University of California Los Angeles, Los Angeles, California, United States of America; 3 Nanjing Center for Disease Control and Prevention, Nanjing, Jiangsu, China; 4 Monitoring and Evaluation Unit, The Global Fund to Fight AIDS, Tuberculosis and Malaria, Geneva, Switzerland; Hopital Bichat Claude Bernard, France

## Abstract

HIV and AIDS incidence in China is high among men who have sex with men (MSM) and ours was one of few studies in China to evaluate the role of HIV risk reduction counseling and testing. Respondent-driven sampling (RDS) was used to recruit 430 MSM. Participants were followed up at 6, 12 and 18 months to evaluate behavioral changes after counseling to reduce risk behaviors. At baseline, HIV prevalence was 4.7%, whereas HIV incidence was 5.2 per 100 person-years. The incidence was 3.8 during six to 12 months, and 1.1 during 12 to 18 months. During the study period, the reported unprotected anal intercourse (UAI) significantly decreased from 60.9% to 42.9%. The proportion of participants who had one or no partner significantly increased from 40.9% to 48.0%. The study also found that some risk behaviors decreased between baseline and 12 months, followed by a slight increase between 12 and 18 months. Reductions in UAI can be achieved through counseling and testing, but may wane over time. Future programs should consider HIV risk-reduction counseling and testing for interventions in MSM in China.

## Introduction

The proportion of HIV infections and AIDS cases is increasing among men who have sex with men (MSM) in China. [1]–[7] It is estimated that among all the cases, new HIV infections in MSM increased from 12.2% in 2007 to 32.5% and 29.4% in 2009 and 2011. [8], [9] The number of MSM living in China is approximately 2–8 million, [10] with data from several major urban areas showing a rapid increase in prevalence of HIV among them. For example, the prevalence increased in Shenzhen from 0.9% in 2002 to 2.7% in 2005, [11] from 0.4% in 2004 to 6.5% in 2007 in Beijing, [12], [13] and from 10.4% in 2006 to 16.7% in 2008 in Chongqing. [14], [15] The HIV epidemic in MSM poses a serious threat to the general population, as MSM may also have unprotected sex with women. [16]–[18] This trend underscores the need for implementation and evaluation of interventions to prevent the spread of HIV infection from and within this risk group.

In an extensive meta-analysis of interventions for MSM, the authors concluded that individual-level behavioral interventions are effective if done in a short span of time. [19] This review also suggested that interventions are most successful when efforts are incorporated to promote personal management skills for MSM. [19] It has also been documented that targeted interventions including reducing alcohol, drug use, risk sexual behaviors and other environmental risk factors can be useful in addressing the epidemic among MSM. [20]–[22].

Among several potential intervention models to reduce risk behaviors, the transtheoretical model (TTM) of behavior change has shown promising evidence [23] for smoking cessation, [24] reduced alcohol use [25], [26] and risk sexual behaviors reduction. [27], [28] This model uses past behavior and behavioral intentions to assess willingness to make changes. [29] The five stages of TTM are pre-contemplation, contemplation, preparation, action, and maintenance. [30] Each stage is necessary before the participants can successfully move to the next stage, and the stages cannot be rushed or skipped. [30].

Using TTM as a theoretical guide, HIV risk reduction counseling sessions can be tailored to encourage behavior changes among participants. Counseling sessions are recognized as an important strategy to reduce the risk of HIV spread in high-risk groups. [31]–[33] They are thought to be most effective when they are planned as a collaborative process involving the members of the targeted group. [34] The counseling sessions play a significant role in imparting HIV prevention education, and can clear up misconceptions and misinformation regarding HIV. It is possible that some participants will be concerned about revealing their personal behaviors in the presence of other participants. Therefore, individualized HIV risk reduction counseling sessions provide an opportunity for participants to discuss sexual information and risky behaviors in private. The sessions play a role in preparing participants to positively contribute towards reducing their risk for HIV and other sexually transmitted diseases (STDs). [9] However, few studies have examined the role of counseling in reducing the risk behavior of MSM in China. [35].

In 2008, a nationwide study of MSM was conducted in 62 cities across Mainland China. The objective of the study was to estimate the prevalence of HIV and syphilis in MSM and understand their behavioral risk patterns. Respondent-driven-sampling (RDS) was used for sampling in 10 of these cities, including Nanjing. At the Nanjing counseling centre, we assessed the efficacy of behavioral interventions offered in the form of counseling sessions about preventing acquisition of HIV and syphilis. The study explored risk behaviors and determinants of HIV/syphilis acquisition at baseline in Nanjing, China, and assessed the effectiveness of a counseling session in reducing risk behavior at each follow-up round.

## Materials and Methods

### Recruitment

Our study employed respondent-driven-sampling [36] to recruit participants, using 10 seeds selected by non-governmental organizations (NGO) for MSM in Nanjing. [5] The seeds recruited MSM from bars, bathhouses/spas, restrooms/parks, and the Internet. After being interviewed, each participant was asked to recruit up to three other MSM with numbered coupons. All recruits were asked to present their recruitment coupons. The original seeds were different in terms of income, age, occupation, and cruising areas. Cruising areas are the venues where MSM look for casual sex partners. Each participant received a packet of lubricant and condoms after being interviewed, and was given a prepaid phone card for each person recruited (up to three). The inclusion criteria for our study were having oral and/or anal sex with a man in the past 12 months, currently living in Nanjing, being at least 18 years old.

### Behavioral Measures

A face-to-face interview collected information from participants. In this study, unprotected anal intercourse (UAI) was defined as lack of consistent use of condoms during anal sex with male partners during the past six months. Unprotected vaginal intercourse (UVI) was defined as lack of consistent use of condoms during vaginal sex during the past six months with all female partners. Information was collected regarding details on recruitment patterns of participants, demographic characteristics, knowledge and attitudes about HIV, preventive services, recent sexual behaviors, recreational drug use, and STD-related symptoms and signs. Higher education was defined as having attended junior college or higher. The survey was conducted at the clinic of the Jiangsu Provincial Center for Disease Control and Prevention in Nanjing. All the participants were offered free HIV/syphilis testing.

### Intervention

HIV risk-reduction counseling and testing was implemented for each participant at each round. The results of the HIV and syphilis testing were matched to individual records after they were obtained from Jiangsu CDC, China. After completing the questionnaire, the participants were invited to a separate room for risk-reduction counseling with one of the two counselors who had previous experience in HIV voluntary counseling and testing at the Jiangsu CDC. The counselors spent five to ten minutes reading the responses to the questionnaire for the interviewee to understand his risk behaviors. They obtained more specific information by asking additional questions. Subsequently, the counselor conducted a detailed counseling session with the participant stressing the implications of his risk behaviors. The counselor answered all the questions from participants. The intervention was completed by providing knowledge about ways to reduce specific risk behaviors, including number of sexual partners during the past six months, sexual practices (anal, oral, vaginal intercourse), patterns of condom use, alcohol and/or drug use affecting sexual activity, STDs, HIV, exchange of sex for money, and assumptions about partners’ risk behaviors. Each participant received free condoms and lubricant, and all HIV-positive participants were referred for standard treatment at the Nanjing Second Hospital.

### Follow-up (Cohort)

After the first survey, all of the participants (except those who were HIV-positive, who were referred to HIV/AIDS care programs) were encouraged to attend the surveys conducted at 6, 12 and 18 months after baseline. Gifts worth US $16 were provided as incentive for each follow-up visit to encourage them to participate. The retention rate was defined as participation in successive rounds compared to the initial round (positive participants were removed from the denominator). For example, the retention rate for the third round was calculated as the number of participants who attended the third round divided by the number of HIV-negative participants in the first round minus the number of HIV-positive participants in the second round.

HIV incidence was estimated by using the number of sero-conversions within each follow-up period as the numerator and the cohort’s total person-year exposures to the risk of HIV transmission within each follow-up period as the denominator. For those who sero-converted, half of the follow-up duration (between two follow-ups) was used as their contribution to the total person time at risk of exposure. The Poisson method was used to estimate the confidence interval of the incidence rates.

### Ethics Statement

Signed informed consent was obtained from each of the participants prior to interview, blood collection and intervention at each round of the surveys. Each of the participants had the ability to decline or withdraw from this survey. The questionnaires and written consent document were separately kept in locked cupboards at the study sites, and unrelated persons cannot access on them. The study process and content were approved by the Ethics Committee at Jiangsu Provincial Center for Disease Prevention and Control.

### Serologic Measures

We have described the serologic measures in detail elsewhere. [35] In brief, before the interview, 5 ml of blood was drawn from each consenting participant to be tested for HIV and syphilis. HIV antibodies were screened using a rapid test (Acon Biotech Co. Ltd). Early studies conducted in China had already successfully used this test. [37] If the result was positive, a western blot was performed to confirm HIV antibody positivity. Syphilis antibodies were screened using Rapid Plasma Reagin (RPR) and confirmed by Treponema Pallidum Particle Agglutination Assay (TPPA). Syphilis positivity was defined as “current” when both TPPA and RPR were positive.

### Data Analysis

Data were double entered using EpiData 3.0. [38] Logic checking was used to clean the data. We used the Respondent Driven Sampling Analysis Tool (RDSAT) version 5.6 [39] to calculate the population adjusted point estimates and 95% confidence intervals of HIV and syphilis at baseline. The RDSAT model was adjusted for social network size and recruitment patterns. Netdraw version 2.119 [40], a program for visualizing social networks graphic distributions, was used to define the recruitment chains of RDS. Generally, the nodes in the graph reflect the participants, while the arrows reflect the recruitment relationship (the participants at the head of the arrows were recruited by the participants at the end of the arrow). Each seed had a separate chain. Trend analysis was used to assess trends in behavior changes over time for the follow-up. Trend analysis and basic description analysis were conducted using Statistical Analysis Software version 9.1 [41] and the threshold for statistical significance was set at *P* value less than 0.05. To test the efficacy of HIV risk-reduction counseling and testing, sensitivity analysis was conducted in this study. In the sensitivity analysis, we removed the prevalent participants and incidence participants from initially from the baseline and at each subsequent follow-up, and analyzed only HIV negative during the whole follow up period.

Possible effect of loss to follow-up was assessed further by assuming that those who dropped out were different from those who stayed in the study, and that those people did not change their behavior even after dropping out. To estimate this effect, we repeated the analysis by imputing the prior values from last visit for those who were lost to follow-up. Thus, we assumed the scenario where the intervention would have had the least effect in changing the participants’ behavior. Therefore, if we still detected an effect of intervention in this “worst-case scenario”, we could safely claim the intervention was not due to selective loss to follow up.

## Results

The study was conducted between May 2008 and January 2010. Four hundred and thirty participants (including the 10 seeds) completed the first survey. Only 33.1% (427) of the 1,289 distributed coupons were returned. Table 1 presents the demographic characteristics of the participants. About 70.2% of the participants were 21 to 35 years old, about 28.9% (124 participants) were students, but only 18.8% were married. Two-thirds had higher education.

**Table 1:** Demographic characteristics of MSM at baseline in Nanjing, China, 2008 (n = 430).

	Sample (n)	Crude %	Adjusted % (95%CI)		Sample (n)	Crude %	Adjusted % (95%CI)
**Age**				**Education level**			
* ≤20 years*	49	11.4	11.5 (7.9, 15.9)	*Illiterate or elementary school*	5	1.2	0.7(01, 1.9)
* 21–35 years*	302	70.2	59.5 (62.5, 75.5)	*Junior high school*	38	8.8	15.5(9.3, 21.1)
* 36–50 years*	71	16.5	16.6 (11.2, 22.8)	*Senior high school or technical secondary school*	99	23.0	25.4(20.3, 31.4)
* 51 or older*	8	1.9	2.4 (0.5, 5.2)	*Junior college or higher*	288	70.0	58.3(50.3, 66.4)
**Marital status**				**Residence**			
* Single, divorced or widowed*	349	81.2	73.0(65.6, 80.0)	*Nanjing*	251	58.4	47.9(41.5, 54.9)
* Married*	81	18.8	27.0(20.0, 34.4 )	*Not in Nanjing*	179	41.6	52.1(45.1, 58.5)
**Occupation**				**Monthly income (RMB)**			
* Student*	124	28.9	18.5(12,22.2)	*≤2000*	137	31.8	25.4(18.7, 31.2)
* Worker*	22	5.0	8.4(5,15)	*2001–4000*	189	44	48.1(42.3, 55.7)
* Staff*	89	20.7	26.7(20.3,32.5)	*4001 and above*	104	24.2	26.5(20.5, 32.3)

At the baseline survey, crude HIV and syphilis prevalence rates were 4.7% and 11.9%. The adjusted HIV and syphilis prevalence rates were 6.6% (95% CI: 3.0–10.4) and 12.6% (95% CI: 8.1–18.3), respectively.

**Figure 1:**
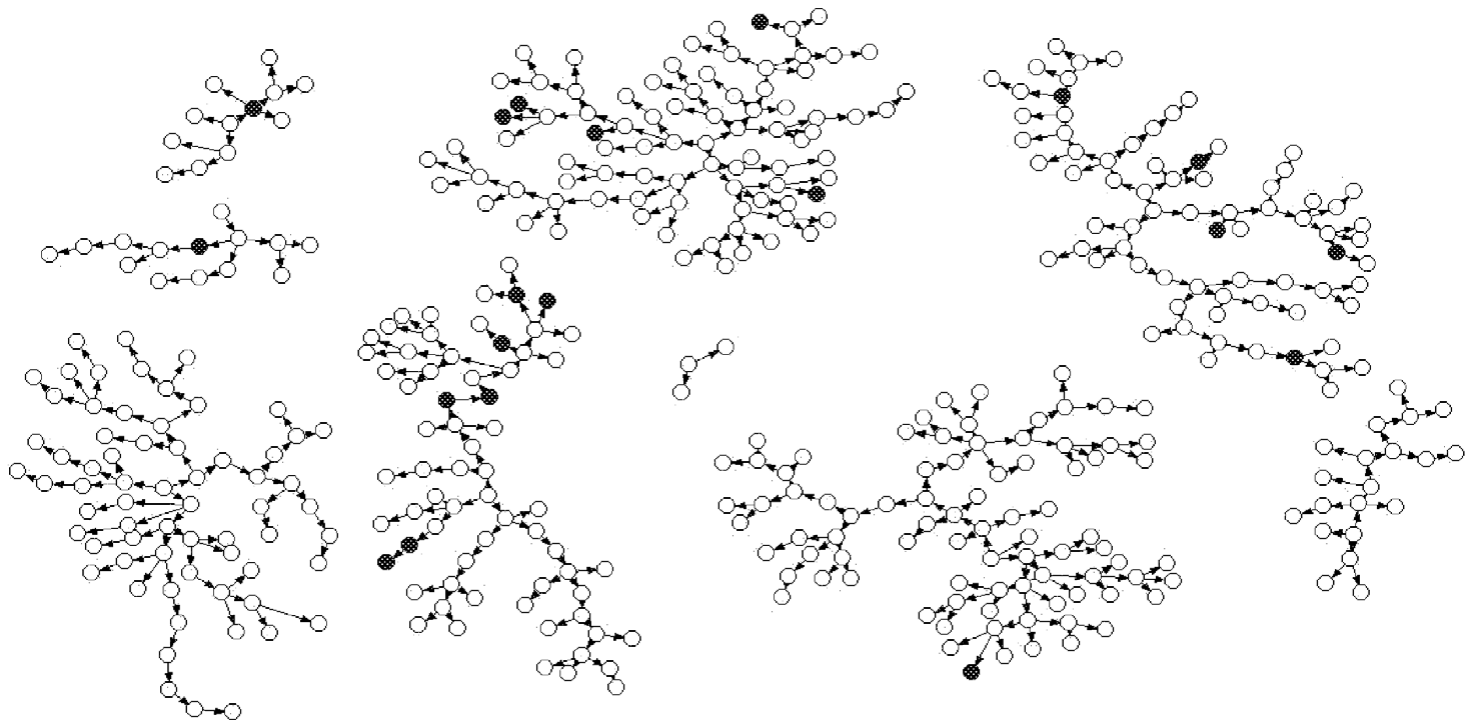
Respondent-driving sampling for cross-recruitment of MSM. Black circles represent HIV-positive and white circles represent HIV-negative participants. One seed that did not recruit any other participants and was dropped from this figure.

Figure 1 shows the recruitment networks at the baseline survey and the waves and networks of participants, as well as the distribution of HIV-positive participants among all the participants. Convergence was reached between the sample composition (4.7% with HIV) and the equilibrium sample composition (4.9% with HIV) (Table 2). Table 2 also demonstrates that the network size (average 30.4) for HIV-positive participants was much larger than for HIV-negative participants (average 19.8).

**Table 2:** Reported rates and status of HIV testing among MSM, in Nanjing, China, 2008 (n = 420*).

Variables	HIV status of participants	
	HIV-positive	HIV-negative
Number of participants recruited by HIV-positive participants (%)	2 (12.5%)	14 (87.5%)
Number of participants recruited by HIV-negative participants (%)	18 (4.5%)	386 (95.5%)
Estimated network sizes	30.4	19.8
Sample proportion, P_s_	0.04	1.0
Equilibrium proportion, P_e_	0.04	1.0
RDS-adjusted proportion, P	0.07	0.9
Absolute discrepancy between P_s_ and P_e_	0.002	
Standard error of P	0.02	0.02
RDS-weight	1.4	1.0
Homophily	0.06	0.3

* When analyzing the total distribution of recruitment between HIV-positive and -negative participants, the RDSAT dropped the 10 seeds from the analysis automatically, since the seeds were selected by researchers, not recruited by participants.

**Figure 2:**
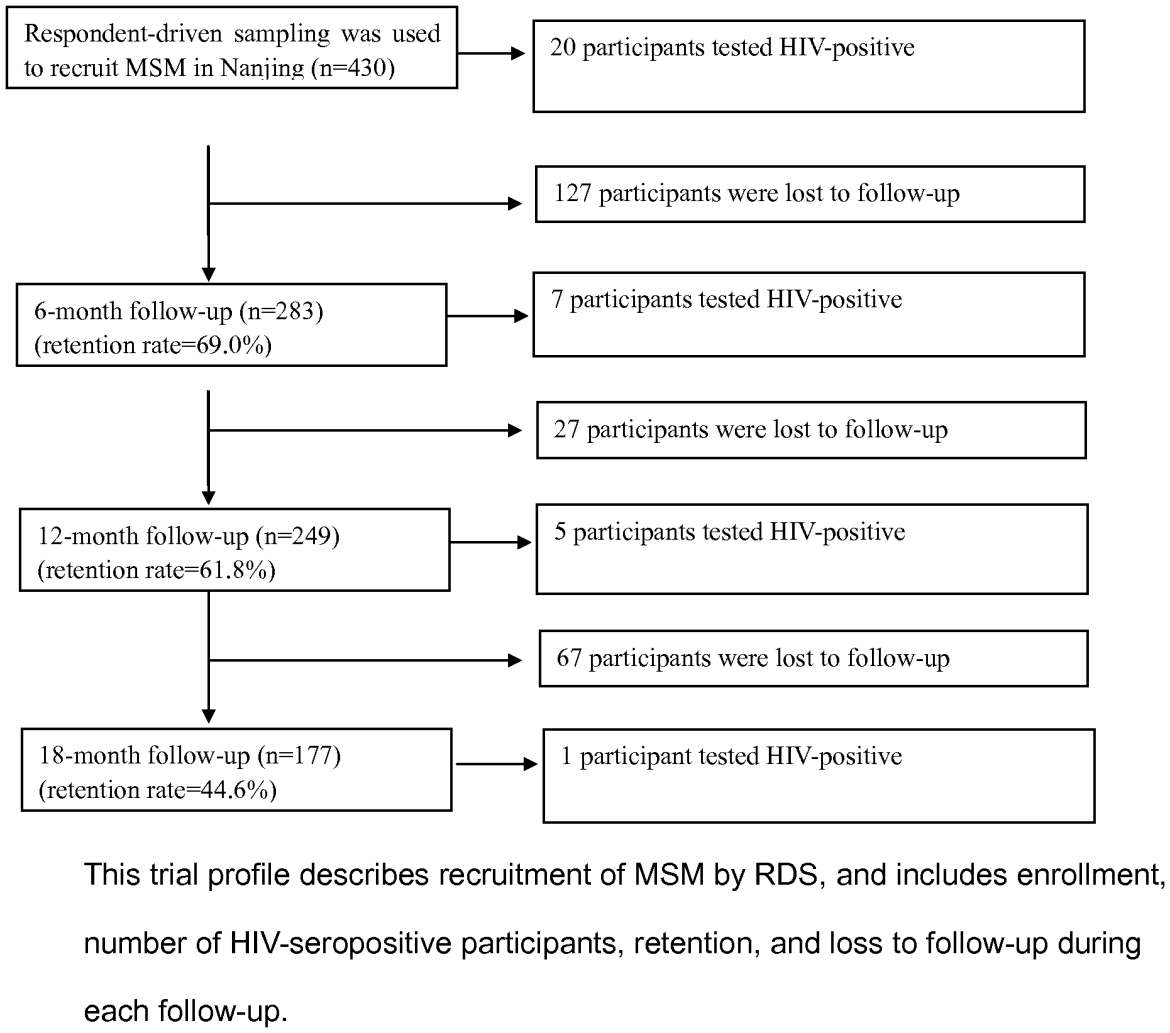
Enrollment, follow-up, and outcome of the participants. This trial profile describes recruitment of MSM by RDS, and includes enrollment, number of HIV-seropositive participants, retention, and loss to follow-up during each follow-up.

**Figure 3:**
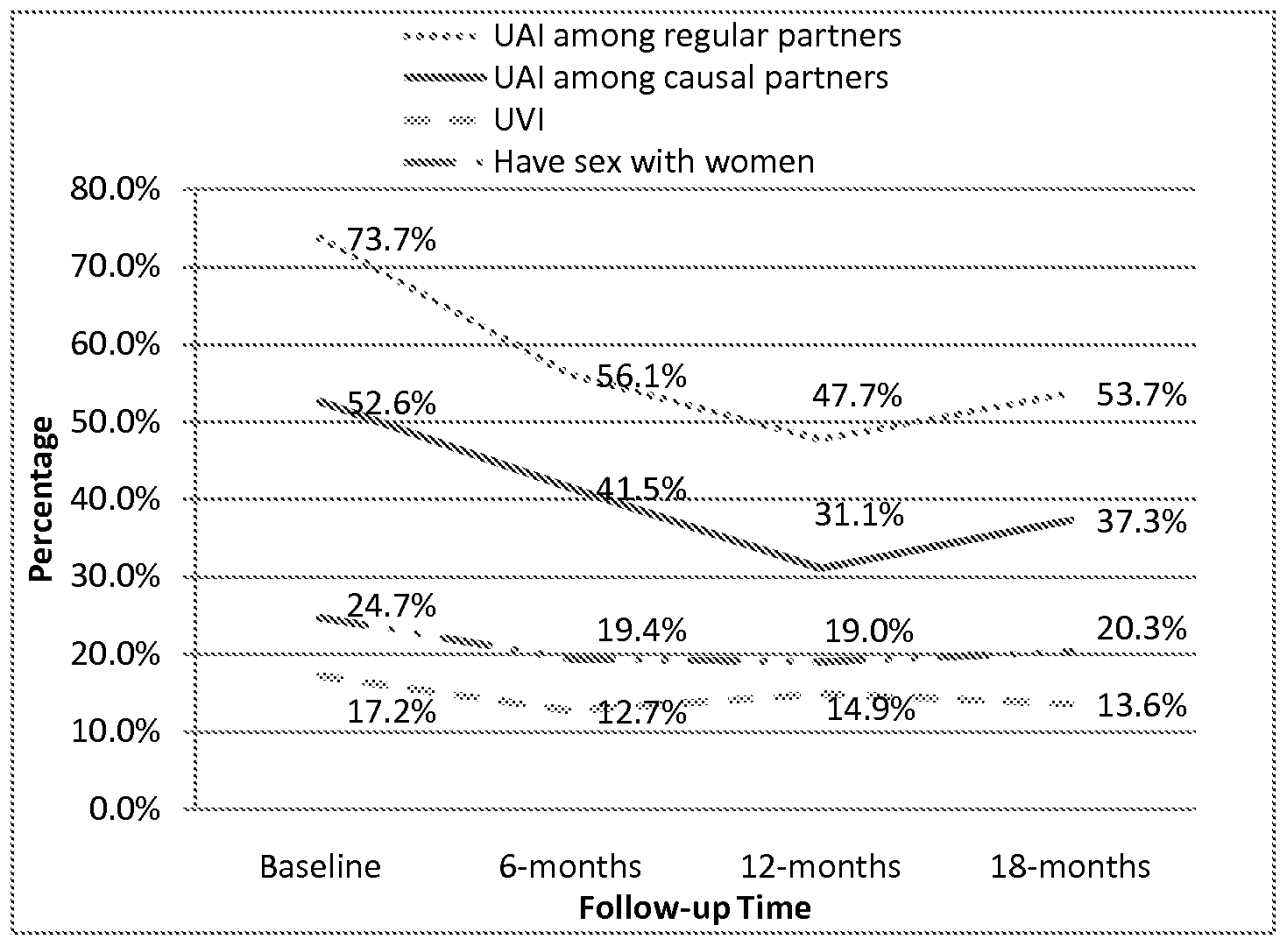
The trend of UAI among regular and casual partners and UVI with women. This figure indicates the trend of high-risk behaviors among regular and casual partners, and the percentage who also would have had sex with women. The greatest decreases in risk behaviors were observed between baseline and the initial six months.

Figure 2 shows recruitment of MSM by RDS, number of HIV-seropositive participants, retention rates, and loss-to-follow-up during each follow-up. The retention rate at six months was 69.0%, 62.2% at 12 months, and 44.6% at 18 months. There were 20, 7, 5 and one HIV-positive participant(s) at baseline, six months, 12 months and 18 months, with an HIV incidence rate at each round of 5.2 (95% CI: 2.1–10.1), 3.8 (95% CI: 1.2–8.8), 1.1 (95% CI: 0.03–6.1) per 100 person-years, respectively, while overall HIV incidence was 3.6 (95% CI: 1.9–6.2) per 100 person-years. At baseline, 40.9% of participants reported that they had no more than one male partner (either regular or casual) in the past six months, which increased to about 48.0% by the end of the study (*P*-value for trend 0.03; Table 3). Also, 60.9% of the participants reported UAI in the past six months at baseline, which decreased to 42.9% by the end of the study (*P*-value for trend <0.001). The results were statistically significant for increased sexual encounters with regular partners and decreased sexual encounters with casual partners (*P*-values for trend 0.02 and <0.001). Table 3 explains the results of sensitivity analysis, which imply similar results as compared to inclusion of HIV positive participants as mentioned above. Figure 3 presents the trend of UAI among regular and casual partners, and the percentage who also would have had sex with women. The percentage engaging in UAI with regular and casual partners decreased between baseline and 12-months; however, it increased between 12 and 18 months. There was not much change in the proportion of MSM having sex with women and UVI. The greatest decreases in risk behaviors were observed between baseline and the initial six months.

**Table 3:** The trend of HIV-related knowledge and reported risk behaviors of the participants in Nanjing, China, 2008–2010.

Variables	Trend analysis					Sensitivity analysis				
	Baseline (N = 430)	six months (N = 283)	12 months (N = 249)	18 months (N = 177)	*P* for trend	Baseline (N = 397)	six months (N = 270)	12 months (N = 243)	18 months (N = 176)	*P* for trend
**Knowledge**	90.2%	95.8%	94.8%	94.9%	0.01*	91.0%	95.9%	95.0%	94.9%	0.04*
**One or no partner**	40.9%	47.7%	52.4%	48.0%	0.03*	41.5%	48.1%	52.1%	47.7%	0.06
**UAI (unprotected anal sex)**	60.9%	49.1%	39.9%	42.9%	<0.001*	59.5%	49.6%	40.1%	42.6%	<0.001*
**UVI (unprotected vaginal sex)**	17.2%	12.7%	14.9%	13.6%	0.4	17.0%	12.2%	14.5%	13.1%	0.3
**Had anal sex with a man in the** **past six months**	88.1%	90.1%	81.8%	85.1%	0.03*	88.0%	90.0%	83.8%	85.1%	0.2
**Paid for sex in the past six months**	5.3%	3.5%	2.8%	1.1%	0.09	5.7%	3.7%	2.9%	1.1%	0.06
**Was paid by another man to have** **sex in the past six months**	6.3%	1.1%	0.8%	0.0%	<0.001*	6.3%	1.1%	0.8%	0.0%	<0.001*
**Had sex with women in the past** **6 months**	24.7%	19.4%	19.0%	20.3%	0.2	24.8%	19.3%	18.2%	19.9%	0.2
**Had sex with regular partner in** **the past six months**	66.2%	73.3%	78.2%	73.3%	0.02*	65.1%	73.3%	78.3%	73.2%	0.006*
**UAI with regular partner in the** **past 6 months**	73.7%	56.1%	47.7%	53.7%	<0.001*	72.9%	56.2%	47.0%	53.3%	<0.001*
**Had sex with casual partner in** **the past six months**	66.8%	62.4%	47.6%	57.8%	<0.001*	66.8%	62.1%	47.8%	58.2%	<0.001*
**UAI with casual partner in** **the past six months**	52.6%	41.5%	31.1%	37.3%	0.001*	51.1%	43.0%	31.5%	37.3%	0.007*
**Last sexual partner for anal sex** **was a regular partner**	54.1%	62.4%	68.0%	64.7%	0.005*	54.3%	62.1%	68.0%	64.4%	0.008*

* Significant differences between groups.

We tested whether the results would change after accounting for loss to follow-up by imputation, by assuming those who dropped out did not change their risk behaviors. The results are provided in Table 4, which indicates that even after imputation, the decrease in UAI among participants was significantly reduced from 60.9% to 46.2% (*P*-value for trend <0.001), and engaging in UAI with regular and casual partners significantly decreased from 73.7% and 52.6% to 57.4% and 38.9%, respectively. The trend analysis with imputation indicated decreased risk behaviors between baseline and 12 months, but an increase between 12 and 18 months.

**Table 4:** The trend of HIV-related knowledge and reported risk behaviors after imputation in Nanjing, China, 2008–2010.

Variables	Baseline(N = 430)	six month(N = 410)	12 months(N = 403)	18 months(N = 398)	*P* fortrend
**Knowledge**	90.2%	93.7%	93.8%	94.0%	0.1
**One or no partner**	40.9%	45.4%	47.9%	46.2%	0.2
**UAI (unprotected anal sex)**	60.9%	50.2%	45.9%	46.2%	<0.001*
**UVI (unprotected vaginal sex)**	17.2%	15.4%	16.4%	16.3%	0.9
**Had anal sex with a man in the past 6 months**	88.1%	89.3%	86.1%	84.8%	0.2
**Paid for sex in the past six months**	5.3%	3.6%	3.1%	2.3%	0.2
**Was paid by others to have sex in the past six months**	6.3%	5.6%	5.4%	5.5%	0.9
**Had sex with women in the past six months**	24.7%	23.7%	22.6%	23.1%	0.2
**Had sex with regular partner in the past six months**	66.2%	69.7%	72.9%	71.2%	0.2
**UAI with regular partner in the past 6 months**	73.7%	58.8%	53.7%	57.4%	<0.001*
**Had sex with casual partner in the past six months**	66.8%	64.2%	56.2%	61.1%	0.02*
**UAI with casual partner in the past six months**	52.6%	41.3%	38.0%	38.9%	0.005*
**Last sexual partner for anal sex was a regular partner**	54.1%	67.9%	61.7%	60.8%	0.2

* Significant differences between groups.

## Discussion and Conclusions

Overall, our study found that reported UAI significantly decreased, from 60.9% to 42.9%, during the study period. Further, UAI with casual and regular partners in the past six months was significant reduced by 15.3% and 20.0% respectively. Similar results for UAI were documented in an randomized control trial by Chun Hao in China in 2008. [35] Comparing with other studies used risk-reduction counseling as intervention strategy in MSM, the results of our study were similar to the findings of the EXPLORE randomized controlled study (our study followed the similar intervention protocol as this study), which found an overall 20·5% UAI reduction in the intervention group. [20] Also, compared to one study conducted in Auckland, New Zealand, [42] our study found much higher UAI reduction rate. However, these reductions were lower than another study conducted in San Francisco, USA. [43] Also, this is the first exploration of whether reductions in UAI occur in MSM who undergo private HIV risk reduction counseling in China. This is also the first cohort study examining risk reduction behaviors in MSM in China.

The results of our study also showed that the proportion of participants who reported one or no partner in the past six months significantly increased. To our knowledge, this is the first time that HIV risk reduction counseling was tested in China as an intervention for reducing numbers of sexual partners. In addition, our study demonstrated that the proportion reporting sex with regular partners in the past six months increased, while the proportion reporting sex with casual partner decreased. There was a decreasing trend of some risk behaviors from baseline to the 12-month follow-up, but not between 12 and 18 months, suggesting waning effectiveness of counseling and testing.

The underlying transtheoretical model of behavior change may explain some of the failure in achieving complete reduction of risk behaviors. According to this theory, there are five stages for behavior change, including pre-contemplation, contemplation, preparation, action, and maintenance. Achieving change in behavior is arduous, as each stage is necessary before moving on to the next. These stages cannot be hurried or skipped. [44] This process can take a long time and may involve cycling back through earlier stages before moving on to the next stage.

Initiation and maintenance of behavior change can be very difficult. Successful behavior change requires people to be motivated to continually sustain positive change over time. Our results are in conformity with existing evidence, which suggests that initiation of behavior change is easier than maintenance. [45]–[47] It is also possible that during the follow-up period, negative reinforcement of risky behavior occurred, which we were unable to identify. [36] It might be contended that the results are due to HIV participants, who may have higher risk behavior. However, the results of sensitivity analysis indicated that results did not change much despite exclusion of HIV participants supporting the conclusion that the behavior changes of the participants are mainly due to the intervention and testing.

There are other important results from this study. The crude HIV and syphilis prevalence at baseline were 4.7% and 11.9% respectively. These prevalence estimates indicate lower burden than compated to other cities in China. [13], [14] The higher rates of newly acquired infection and lower prevalence suggest a high fatality in MSM. The high incidence in Nanjing (overall 3.6 per 100 person-years during the entire follow-up) calls for planning intervention programs targeting MSM in Nanjing. Reduction of HIV incidence in Nanjing during the follow-up period (from 5.2 per 100 person-years to 1.1) confirmed the reported behavior changes.

The strengths of the study include its use of RDS to access members of the otherwise hard-to-reach populations in a demographically diverse sample. [36] This is the main difference between our study and some previous studies. Convenience sampling (or targeted sampling), and time-space sampling are the main sampling strategies that were used by the earlier studies [20], [48]–[50]. However, these methods often fail to let the researchers to make accurate estimates about a hidden population. [51] Also, a report by WHO demonstrated that standard sampling and estimation techniques do not have the ability to monitor the behavior and HIV sero-prevalence of at-risk subpopulations, such as MSM. [52] RDS potentially address this problem even in the studies with lower response rate and loss to follow up. By combining of these two methods (RDS and standard HIV risk-reduction-counseling and testing), the study aids in updating current knowledge about the effectiveness of HIV risk-reduction-counseling and testing. In addition, this is the first time in China that follow-up study is done using RDS for initial recruitment and hence important to report in addition to exploring further whether further studies demonstrate the success of HIV risk-reduction and counseling in China.

The use of biological markers of HIV and syphilis infection is also a potential strength of our study. A follow-up study monitored the change of risk behaviors of the participants. Also, compared with other studies, including several studies targeting MSM, [23]–[26], [42], [53], [54] our study had strengths of having longer intervention period and follow up time. Besides these, all the HIV sero-positive participants were referred to the standard care and treatment program running by Chinese CDC. All diagnosed persons with syphilis were referred and treated at the STD clinic at our study site.

The major limitations of our study were the low response rate and high rate of loss to follow-up. The loss to follow up, in the initial period from baseline to 6 months might bias the results, which in turn may potentially affect the validity of our results. However, this is possible only if the loss to follow up was differential (the loss to follow up was associated with exposure and outcome). [55] It might be possible that those who were lost to follow up might have higher HIV incidence rate or UAI rate than those consistently participated, in which case the loss to follow-up might have overestimated the association that we measured. However, it is more likely that it is not associated outcome of interest as the participants migrated out of the study due to their temporary engagement in Nanjing due to their occupation or otherwise, in which case, the loss to follow-up is non-differential and hence may or may not underestimate the results. Without additional information, we cannot conclusively predict the direction of the bias nor can this bias be adjusted in conventional analysis. Future studies might provide more evidence regarding the behavior of people in LMICs and how their loss to follow-up might affect the hypothesis of interest. There are several potential reasons for the higher lost to follow up rate. First, about one-fifth of the participants were students who graduated from university and subsequently moved away from Nanjing during the study period, and were not accessible. The number of students at the end of the study was only 30, compared to 124 students at the beginning of follow-up. Second, lack of adequate funding limited our ability to continue to trace the participants for further follow-up. Frequent migration between different cities makes tracking of participants a challenging task (among the 127 participants who were lost to follow-up at the six months follow-up, 67 had already moved to other cities and could not be reached). [56].

We performed imputation analysis to explore the sensitivity of results to loss to follow-up by assuming that the participants lost to follow-up continued the same behaviors as in the immediate previous round of follow-up. This strategy assumes that there was no effect of the intervention on behavior change among those lost to follow-up, and thus biases the results towards the null. Behaviors were self-reported and were thus subject to desirability bias. However, the drop in HIV and syphilis incidence suggests that the reported changes were real.

Another limitation of our study was that only 427 (33.1%) of the 1289 coupons distributed were returned. Even though the low response rate is common in such studies and is consistent with other RDS surveys, [57], [58] the low response rate may result in limiting generalizability to MSM population. The participants who attended the survey may be different from those who unwilling to come. Also, we could not address this by data analysis as no information was available regarding those who refused.

The absence of a suitable cohort of control participants is a limitation for our study. It is possible that sources of information other than counseling might be available, such as national media campaigns or learning from friends. We also could not evaluate the impact of this program on risk behaviors because we lacked a control group who did not receive counseling and testing. We therefore advice caution for making causal inference from our study, as we could not rule out the other probabilities that lead to the behavior change of the participants. Randomization is the gold standard and our inability to have such comfort is another limitation of our study due to which uncontrolled confounders might have affected our results. We have included all the known confounders; however, might have missed out those not identified by prior literature or based on our contextual knowledge regarding their role in possible causal association.

We used certain methods to minimize the bias. Four specially trained interviewers used a standardized protocol consistently throughout the study. Well-trained CDC staff conducted the interviews and interventions. Quality assurance was enhanced by having two separate workers check each questionnaire carefully. We analyzed loss to follow-up by performing imputation analysis when assuming that there were no significant changes in behavior after loss to follow-up.

Despite the limitations, we conclude that HIV/STD risk-reduction counseling and testing is an effective method to reduce the risk behaviors of MSM and prevent new HIV/STDs in the short term, but may require continued intervention to assure maintenance. Also, the higher HIV/syphilis prevalence and incidences in our study call for more surveillance programs to monitor the future HIV/syphilis epidemic as well as the risk behaviors of MSM in Nanjing. It is important to screen all the MSM for syphilis and ensure treatment to affected persons irrespective of risk reduction interventions.
